# Process and Strategies for Implementing an Antenatal Psychosocial Clinical Decision Support System Within an Inter-Organisational Care Context: The Born in Belgium Professionals Platform

**DOI:** 10.3390/healthcare14111508

**Published:** 2026-05-29

**Authors:** Kelly Amuli, Kim Decabooter, Caroline Germanes, An-Sofie Van Parys, Sabine Verschelde, Emilie Saey, Manon Moulin, Pieter Cornu, Katrien Beeckman

**Affiliations:** 1 Nursing and Midwifery Research Group (NUMID), Faculty of Medicine and Pharmacy, Primary Care (PRIM), Universitair Ziekenhuis Brussel (UZ Brussel), Vrije Universiteit Brussel (VUB), Laarbeeklaan 101-103, 1090 Brussels, Belgium; 2 Born in Belgium Professionals & Universitair Ziekenhuis Brussel, 1090 Brussels, Belgium; kim.decabooter@uzbrussel.be (K.D.); caroline.germanes@uzbrussel.be (C.G.); an-sofie.vanparys@uzbrussel.be (A.-S.V.P.); sabine.verschelde@uzbrussel.be (S.V.); emilie.saey@uzbrussel.be (E.S.); manon.moulin@uzbrussel.be (M.M.); 3 Research Centre for Digital Medicine, Vrije Universiteit Brussel, Universitair Ziekenhuis Brussel, 1090 Brussels, Belgium; pieter.cornu@uzbrussel.be; 4 Department of ICT, Universitair Ziekenhuis Brussel, 1090 Brussels, Belgium; 5 Verpleeg- en Vroedkunde, Centre for Research and Innovation in Care, Midwifery Research Education and Policymaking (MIDREP), Universiteit Antwerpen, 2610 Antwerp, Belgium

**Keywords:** Clinical Decision Support Systems, pregnancy, implementation science, digital health technology, psychosocial care, integrated care, EPIS framework, ERIC taxonomy, implementation process, implementation strategies

## Abstract

**Highlights:**

**What are the main findings?**

- The implementation process of an antenatal psychosocial clinical decision support system (CDSS) unfolded across the Exploration, Preparation, Implementation, Sustainment (EPIS) phases.
- The implementation process was shaped by coordination roles, system-level support, and organisational leadership. Bridging factors, primarily enacted by implementation agents, were central throughout the process by linking policy-level decisions, organisational contexts, and practical use of the CDSS.
- Stakeholder engagement and iterative approaches were the most frequently described ERIC implementation strategies.

**What are the implications of the main findings?**

- Implementation strategies such as stakeholder relationship-building, iterative evaluation, training, and interactive assistance were essential to support adoption, scale-up, and long-term sustainability in complex healthcare settings.

**Abstract:**

**Background/Objectives**: Despite ongoing innovation, few interventions—including Clinical Decision Support Systems (CDSS)—are successfully integrated into routine care. Understanding the process through which innovations are implemented is therefore essential for advancing practice and research. In perinatal settings, evidence on how CDSS implementation unfolds and which strategies support adoption, scale-up, and sustainment remains limited. This study aimed to understand the implementation process, key determinants and implementation strategies of a shared antenatal psychosocial CDSS (i.e., the Born in Belgium Professionals [BIB-Pro]) implemented in a real-world, cross-sectoral perinatal care setting. **Methods**: A qualitative exploratory case study was conducted between January and March 2025. Data included semi-structured interviews with all seven implementation agents, document analysis of the implementation plan. Directed content analysis was applied using the Exploration, Preparation, Implementation, Sustainment (EPIS) framework to categorise contextual determinants and the ERIC taxonomy to classify implementation strategies. Data were synthesised across the four EPIS phases. **Results**: The implementation process unfolded across all EPIS phases, showing a shift in responsibility from the policy level to the implementation team and healthcare organisations. Implementation was shaped by key determinants across multiple levels: (1) the bridging functions by the BIB-Pro implementation agents connecting policy, innovation, and organisational practice; (2) the system-level leadership and funding by the National Institute for Health and Disability Insurance that enabled initiation and sustainability; and (3) the multilevel stakeholder involvement and inter-organisational collaboration across care settings. In addition, the personal attributes of implementation agents—accessibility, active listening, adaptability, and persistent follow-up—were also identified as relevant factors in the implementation process. Across the implementation process, a broad range of implementation strategies was identified. The most prominent ERIC strategies were developing stakeholder interrelationships, evaluative and iterative strategies, engaging stakeholders, training and educating stakeholders, and providing interactive assistance. Barriers encountered during the implementation process included fragmented care networks, inconsistent regional referral structures, legal uncertainties, and variable digital readiness. In response to these challenges, implementation strategies were applied to support collaboration, clarify procedures and provide targeted support. **Conclusions**: This study provides insight into how a CDSS was introduced, scaled, and sustained across complex multiple Belgian perinatal care settings. Strong bridging functions, stakeholder interrelationships, iterative evaluation, and system-level support were key factors throughout the implementation process. Across all phases, stakeholder interrelationship strategies and evaluative and iterative strategies were the most prominent and consistently applied, supporting stakeholder engagement and sustained use of the platform. These findings offer actionable guidance for implementing digital tools in multi-organisational and multi-level contexts within perinatal care and other healthcare settings.

## 1. Introduction

Despite the development of numerous innovative interventions, few are successfully integrated into clinical practice in a timely or sustainable manner [1]. As Bauer et al. (2020) emphasise, even an intervention with proven effectiveness can fail in real-world settings due to influencing factors [2]. This is supported by the systematic review and meta-analysis by Kouri et al., which found that the uptake of Clinical Decision Support Systems (CDSS) is not primarily determined by the technology or content itself, but rather by the availability and quality of patient data, as well as the surrounding context and implementation strategy [1].

These findings highlight the importance of implementation science, which examines methods and strategies supporting the uptake of evidence-based practices and research findings into routine care. It focuses on understanding the implementation process, determinant factors (i.e., influencing contextual factors), and implementation strategies to promote effective adoption, scale-up, and sustainability [3,4]. Recent implementation research has primarily aimed to identify key determinants of implementation [5]. However, identifying determinants alone provides limited guidance on how implementation should be organised, coordinated, and sustained in real-world settings. The implementation process, as well as implementation strategies, have been less reported in implementation science, despite being essential to understand implementation in real-world settings [6,7,8]. As Santema et al. highlight, determinant frameworks provide limited “how-to” guidance for translating implementation knowledge into practice. While such frameworks are valuable for identifying factors that influence implementation, they offer limited insight into the underlying processes through which implementation unfolds over time [9]. A process-level perspective that provides contextual determinants and concrete implementation actions is more informative to understand how implementation occurred in real-world settings.

This is particularly relevant in complex healthcare systems characterised by fragmented structures and multiple interdependent organisations and multi-level structures [10], where implementation requires coordination across sectors and stakeholders, as described by Santema et al. CDSS inter-organisational implementation research [9].

In this context, valuable insights can be drawn from a shared (i.e., collaborative) antenatal CDSS, the Born in Belgium Professionals (BIB-Pro) platform [11], which has been implemented in Belgian perinatal care settings. The platform supports care providers in the detection, follow-up, and referral of pregnant women facing psychosocial difficulties. Its implementation takes place within a complex context, characterised by multiple cross-sectoral care settings (i.e., medical and social care settings), a fragmented healthcare system with increasing demands for integrated care, and a shift in psychosocial screening practice [12,13]. Previous findings, based on ongoing (unpublished) analyses and information available on the BIB-Pro website [14], indicate adoption and sustained use of the platform over time, particularly in larger medical settings (e.g., hospitals), alongside positive user perceptions [11]. These implementation outcomes (i.e., adoption, acceptability, and early indications of sustainability) serve as indicators of implementation success [15].

Despite previous antenatal psychosocial CDSS studies [16,17,18,19] reporting on performance and users’ perceptions, research on implementation processes, determinants, and strategies remains scarce, particularly for informing broader scale-up. Therefore, this case study aims to examine the implementation of the shared BIB-Pro CDSS platform in Belgium. It seeks to map the implementation process, contextual determinants, and implementation strategies that have facilitated the integration of the platform into routine care of multiple settings. In doing so, it provides more insight into the implementation of a complex digital innovation in a real-world, cross-sectoral perinatal care setting.

## 2. Materials and Methods

### 2.1. Design

We conducted an exploratory case study to examine the implementation process of a CDSS implemented across multiple settings, with the aim of identifying key determinants and implementation strategies. A qualitative study was conducted between January and March 2025 to map the implementation process and strategies used during the dissemination and implementation of the BIB-Pro platform. The study included semi-structured interviews with implementation agents, a document analysis of the implementation plan, and a structured form to validate the mapping against the ERIC taxonomy. This study complies with the Consolidated Criteria for Reporting Qualitative Research (COREQ) checklist and was reported accordingly [20].

### 2.2. Case Description

The BIB-Pro platform is a national initiative of the NIHDI, aimed at supporting care providers with the detection, follow-up, and referral of psychosocial vulnerabilities during pregnancy. The implementation and coordination of the platform are led by a team (called BIB-Pro team) of implementation agents—defined as those responsible for supporting and carrying out the implementation of an intervention within a given context [4]—and in collaboration with other stakeholders. The BIB-Pro team is multidisciplinary and consists of professionals with backgrounds in midwifery, research, psychology, and public health, as well as profiles in project management and implementation coordination.

Since its launch in 2021, the BIB-Pro platform has been implemented across Belgian cross-sectoral perinatal care settings, including hospitals and community care organisations such as social care and primary care [11]. Positive user perceptions have been reported in previous work [11], while additional indications of adoption and sustained use in practice are supported by ongoing (unpublished) analyses and reflected in the diversity and number of partner organisations involved in the platform, as documented on the Born in Belgium Professionals website [14].

### 2.3. Participants

Participants in this study consisted of implementation agents from the BIB-Pro team, who were directly involved in the development, coordination, and implementation of the platform across Belgian care settings. A purposive sampling strategy to include all implementation agents involved in the national roll-out of the BIB-Pro platform, as they were considered key informants regarding the implementation process. Given their central role as bridging actors throughout the implementation process. All seven implementation agents were invited to participate in the study and were approached face-to-face at their workplace for participation. All invited participants agreed to participate, resulting in a total sample of seven participants. No participants refused or dropped out of the study.

We specifically focused on implementation agents as key informants, given their central role as bridging actors throughout the implementation process. They facilitated alignment between policy-level decisions, organisational contexts, and the practical use and development of the CDSS across multiple settings.

### 2.4. Data Collection

We collected multiple sources of data, including semi-structured interviews with implementation agents and document analysis of the implementation plan, aiming for data triangulation to enhance the validity of the findings. In total, we recruited and interviewed all seven implementation agents involved in the national roll-out of the BIB-Pro platform between January and March 2025. Interviews were conducted until data saturation was reached, meaning that additional interviews confirmed existing findings rather than generating new insights.

All interviews were conducted face-to-face at the participants’ workplace and lasted between 45 and 90 min. Interviews were audio-recorded with participants’ consent and transcribed verbatim. Field notes were taken during and after the interviews to capture contextual information and initial reflections. No repeat interviews were conducted, and transcripts were not returned to participants for correction.

### 2.5. Semi-Structured Interview Guide

**Figure 1:**
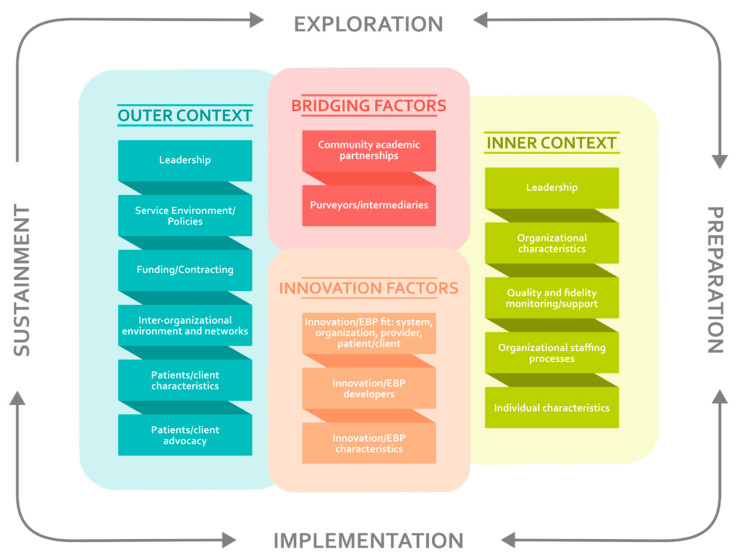
Overview of the EPIS framework adapted from Aarons et al. [6] and Moullin et al. [22]. Legend: The Exploration, Preparation, Implementation, and Sustainment (EPIS) Framework conceptualises implementation as a multi-phase process shaped by four interacting contextual domains: Outer Context (blue), Inner Context (green), Innovation Factors (orange), and Bridging Factors (red). Each domain includes constructs influencing the adoption and sustainment of innovations, such as leadership, funding, organisational characteristics, innovation/EBP fit, and others. Definitions and descriptions of these phases, factors, and constructs can be found on https://episframework.com/ (accessed on 31 March 2026). Figure adapted from Aarons et al. (2011) [6] and Moullin et al. (2019) [22], represented in our own colours and layout (Permission to use: Systematic review of the Exploration, Preparation, Implementation, Sustainment (EPIS) framework, Implementation Science, 14:1, doi: 10.1186/s13012-018-0842-6. Licence: Creative Commons Attribution 4.0 International (CC BY 4.0)).

To map the BIB-Pro implementation process, we developed a semi-structured interview guide (See Supplementary File S1) based on the four phases of the EPIS framework (Exploration, Preparation, Implementation, and Sustainment) (Figure 1). Additionally, to identify the implementation strategies applied, we collected examples of ERIC strategies from the interviews and the implementation plan and mapped them against the ERIC taxonomy using a structured form (See Supplementary File S2).

### 2.6. Selected Frameworks to Map Data

While implementation strategies are often conceptualised as the ‘what’ of implementation (i.e., methods and techniques), the implementation process represents the ‘how’ an intervention is put into practice. Understanding the implementation process and strategies of successfully implemented CDSS might enhance practice, while their classification through standardised frameworks such as EPIS [6], ERIC [21] might advance research by enabling cross-study comparability.

The Exploration, Preparation, Implementation, Sustainment (EPIS) framework provides useful guidance in this regard [6]. It also integrates both internal and external contextual factors that can influence the process. This comprehensive framework helps to understand and structure implementation processes, determinants, strategies, and evaluation [22].

To ensure consistency, comparability, and reproducibility across implementation initiatives, researchers often rely on the Expert Recommendations for Implementing Change (ERIC) taxonomy, which includes 73 discrete strategies [21]. These were later grouped into thematic clusters by Waltz et al. (2015) [23].

#### 2.6.1. The EPIS Framework

The EPIS Framework conceptualises the implementation process through four phases (Exploration, Preparation, Implementation, and Sustainment). The Exploration phase involves identifying existing needs and suitable interventions. The Preparation phase involves planning, adapting, and building capacity for rollout. During the Implementation phase, the chosen intervention is launched, and efforts are made to support adoption and sustained use. Finally, the Sustainment phase focuses on maintaining, monitoring, and optimising the intervention over time. Across these phases, the framework considers four main contextual factors (outer context, inner context, bridging factors, and innovation factors), each represented by a corresponding colour (Figure 1). Each factor includes specific constructs, such as leadership, funding, inter-organisational collaboration and others, that shape how implementation unfolds (Figure 1). Definitions and descriptions of these phases, factors, and constructs can be found on their website https://episframework.com/ (accessed on 31 March 2026).

#### 2.6.2. The ERIC Taxonomy

The Expert Recommendations for Implementing Change (ERIC) taxonomy is a compilation of 73 discrete implementation strategies, developed to standardise the reporting, description, and evaluation of implementation efforts [21]. These strategies are organised into nine conceptually distinct categories as proposed by Waltz et al. (2015) [23] and can be found in Supplementary File S2. In this study, implementation strategies were identified from interviews and the implementation plan and subsequently mapped against the ERIC taxonomy using a structured form. Each identified strategy was classified under its corresponding Waltz category.

### 2.7. Data Analysis

A directed content analysis was performed. All audio recordings were transcribed using Cockatoo software (https://www.cockatoo.com, accessed on 31 March 2026), and coding was performed by a PhD researcher. The analysis followed a structured, framework-based approach.

In the first step, we applied deductive coding based on the constructs of the EPIS framework (e.g., funding, inter-organisational collaboration, leadership). This allowed us to categorise data within the broader contextual factors (i.e., outer context, inner context, bridging factors, and innovation factors) and across the four EPIS phases (Exploration, Preparation, Implementation, and Sustainment) (see Figure 1).

In the second step, identified actions and interventions were deductively coded against the ERIC taxonomy (See Supplementary File S2) to classify discrete implementation strategies, which were subsequently grouped into the higher-level clusters defined by Waltz et al. (2015) [23].

The coding structure was predefined based on EPIS constructs and ERIC strategy categories, which together formed the coding framework used to organise the data.

Data were organised and managed in Microsoft Excel to systematically structure codes, categories, and illustrative quotes. Document analysis of the implementation plan was done for data triangulation to enhance the validity of the findings. For interpretation, the data were structured according to the four EPIS phases to provide an overview of how the implementation process and related strategies evolved over time.

## 3. Results

**Figure 2:**
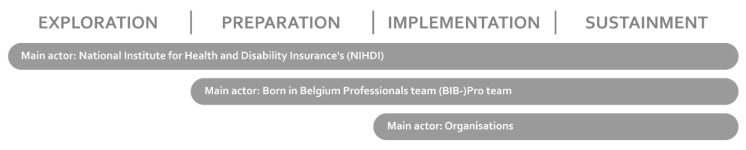
Main stakeholders fostering the implementation of BIB-Pro in each EPIS phase.

All seven implementation agents (IA) were interviewed, and the interviews lasted between 45 and 90 min. Different main stakeholders were identified across each phase of the EPIS framework within the BIB-Pro implementation process, highlighting the transition of responsibility from NIHDI to the BIB-Pro team, and finally to (health)care organisations. Figure 2 illustrates the main stakeholders fostering the implementation of BIB-Pro throughout the successive EPIS phases.

Results below represent the BIB-Pro implementation process, according to the four EPIS phases: Exploration, Preparation, Implementation, and Sustainment. Each phase provides an overview of the present contextual factors (represented by a colour) with their related constructs alongside the applied ERIC implementation strategies in that phase, according to the taxonomy. A detailed representation of these elements is provided in the figures and in Supplementary File S2.

### 3.1. Exploration

**Figure 3:**
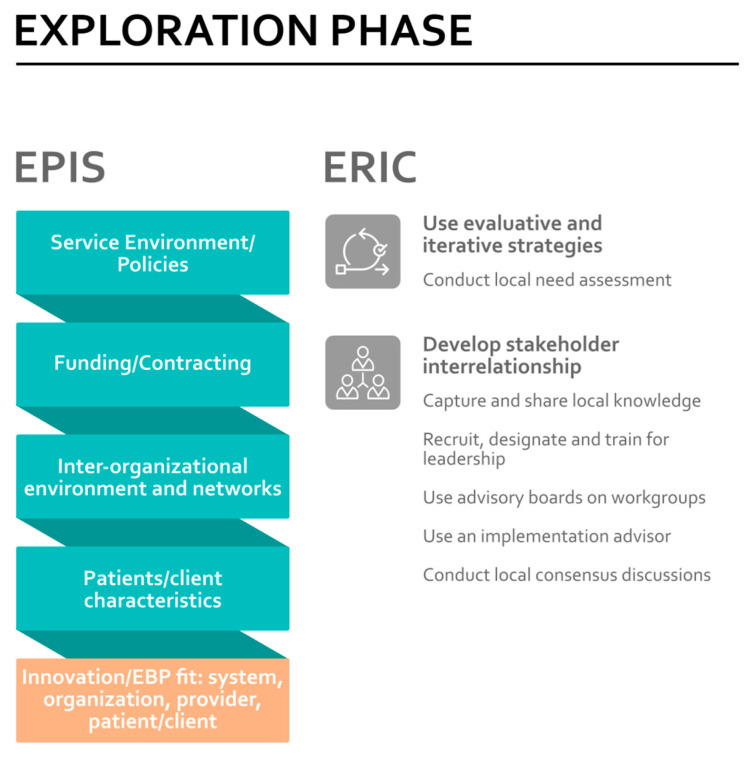
EPIS constructs and ERIC strategies identified in the Exploration phase.

The Exploration phase was initiated by the NIHDI, reflecting the EPIS construct ‘Service Environment/Policies’ (Figure 3), in response to a growing awareness of the need for early detection and tailored support for pregnant women in psychosocially vulnerable situations. This phase was supported through funding and contracting arrangements (EPIS construct ‘Funding/Contracting’), which enabled the project deployment.

Consistent with the EPIS construct ‘Inter-organisational Environment and Networks’, emphasis was placed on integrated care that fosters collaboration among care providers across the care continuum (from community care—including primary care—to hospital services) and communication facilitation through shared Electronic Health Record (EHR).

IA1: “*The White Paper on the Belgian healthcare system, written by the minister of health care, included one chapter dedicated specifically to improving [access to] care for vulnerable pregnant women in Brussels.*” This was the starting point of the project.

In alignment with the EPIS constructs ‘Patients/Clients characteristics’ and ‘Innovation/EBP fit’, attention was given to understanding the needs and identifying the most appropriate intervention. The NIHDI used strategies such as conducting a local need assessment and holding discussions with experts in the field of psychosocial vulnerability during pregnancy (Figure 3). These actions led to an initial concept of the intervention: a free-of-charge shared psychosocial antenatal CDSS designed for care providers.

### 3.2. Preparation

Once the intervention objectives were defined, the Preparation phase began. Barriers and facilitators were identified, and a dissemination and implementation plan was developed. The Preparation phase unfolded in five steps.

#### 3.2.1. Step 1: Getting the Intervention Ready

The NIHDI allocated funding and appointed the BIB-Pro team for the intervention creation and deployment. Reflecting the EPIS construct ‘Funding/Contracting’, the NIHDI established clear, goal- and results-oriented deadlines, with the possibility of project and funding renewal upon achieving predefined objectives and outcomes.

The BIB-Pro team coordinated the development and deployment of the intervention. In their role as Purveyors/Intermediaries (EPIS bridging factors), implementation agents served as a liaison across domains: the inner context, the outer context, and the innovation itself. According to the EPIS framework, they can be described as organisations or individuals providing support or consultation for implementation. Together with the IT developer, they also acted as Innovation/EBP Developers, responsible for creating and refining the intervention to ensure continuous quality improvement.

The BIB-Pro team developed an antenatal psychosocial CDSS, planned its dissemination and implementation. This process addressed Innovation/EBP Characteristics such as adaptability, usability, and legal and technical fit. The team created a screening questionnaire informed by literature, care providers, and experts (71). They co-developed follow-up care pathways and integrated network databases. They ensured the platform’s technical and legal usability and required standards. These actions involved Community–Academic Partnerships, whereby experts, researchers, advocacy groups and care providers collaborated to co-develop the content and structure of the platform. Consistent with the EPIS construct Patient/Client Advocacy, this collaboration ensured that the perspectives and priorities of care providers—as the primary end-users—were represented throughout the design process. This collaborative design thus ensured an Innovation/EBP Fit, aligned with practical realities and user needs.

**Figure 4:**
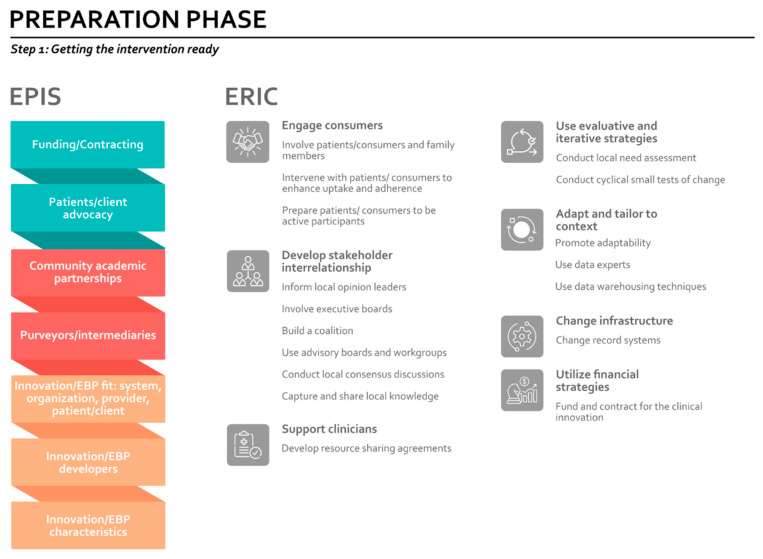
EPIS constructs and ERIC strategies applied during step one of the Preparation phase.

Strategies in this phase (Figure 4) focused on consumer engagement to guide development and foster stakeholder buy-in, complemented by evaluative and iterative approaches such as needs assessments and pilot tests.

#### 3.2.2. Step 2: Preparing Implementation Agents

During this step, the EPIS construct ‘Purveyors/Intermediaries’ was central. The BIB-Pro implementation agents, as Purveyors/Intermediaries, developed and continuously refined a dissemination and implementation plan, with each agent responsible for a specific Belgian region. Implementation followed a phased approach: first engaging organisations with large target groups (i.e., pregnant women), then supporting integration into workflows, and finally involving surrounding networks to promote inter-organisational collaboration.

**Figure 5:**
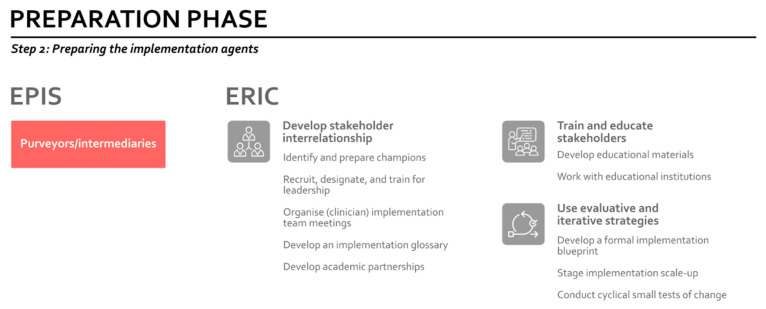
EPIS construct and ERIC strategies applied during step two of the Preparation phase.

Strategies in this phase (Figure 5) included, amongst others, developing a formal implementation blueprint, staging the scale-up, and applying cyclical tests of change.

#### 3.2.3. Step 3: Outreach and Awareness-Raising Among Care Providers

**Figure 6:**
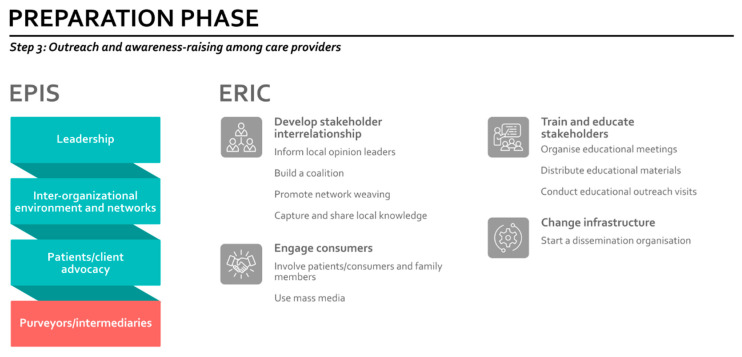
EPIS construct and ERIC strategies applied during step three of the Preparation phase.

During this step, factors within the outer context (Figure 6) were central, particularly the EPIS constructs ‘Leadership’, ‘Inter-organisational Environment and Networks’, ‘Patient/Client Advocacy’, and ‘Purveyors/Intermediaries’ (Bridging Factors).

The BIB-Pro implementation agents, as Purveyors/Intermediaries, acted as advocates for integrated psychosocial antenatal care (patient/client advocacy) by promoting the platform and fostering stakeholder collaboration.

They took leadership in disseminating information about the platform and engaging care providers, professional associations, governmental bodies, (local) (healthcare) managers, etc., through emails, newsletters, social media, the project website, and newspapers. They organised webinars, awareness days, and symposia to present the platform, share updates, and highlight good practices, while also participating in working groups, conferences, and steering committees organised by stakeholders.

Consistent with the EPIS construct ‘Inter-organisational Environment and Networks’, collaboration with experts and organisations with prior experience in implementing similar interventions or maintaining regular contact with care providers was equally important. These partners provided strategic guidance, shared best practices, and fostered key connections—support that was invaluable for preventing and addressing resistance.

As one agent noted: (IA7) “*The two key lessons are: (1) invest in effective communication and (2) build a trusting relationship.*” Another stressed: (IA4) “*If collaboration with external partners isn’t in place, it’s best not to start at all.*”

Strategies applied during this step are summarised in Figure 6, including the use of mass media to raise awareness.

#### 3.2.4. Step 4: Achieving Organisational and Care Provider Adoption

During this step, both outer and inner context factors became crucial. According to implementation agents, adoption of the BIB-Pro platform depended on how organisations and care providers weighed its benefits against perceived impacts on workflows and resources. In addition, peer influence and the involvement or recommendations of external stakeholders also play a significant role in the adoption process.

Consistent with the EPIS constructs ‘Leadership’ (Outer context) and ‘Inter-organisational Environment and Networks’, the team’s key strategy was to involve influential “lead” organisations—such as the NIHDI, governmental organisations that provide social health services for young families, regional community care networks, and influential hospitals—early in the process. These advocates or ambassadors triggered other organisations and professionals to adopt the platform.

IA2: “*We often find that one organisation needs to be sufficiently advanced [in the implementation process]—already underway—before others, particularly in a hospital setting, will follow suit.*”

Conversely, the team faced resistance from one influential regional perinatal body, backed by governmental funding and strong local collaborations. Their opposition created a barrier, as many organisations and care providers were unwilling to adopt the platform unless this body was also on board. Acting as Purveyors/Intermediaries (Bridging Factors), implementation agents aimed to address this resistance through regular meetings and discussions to reach common ground. As one implementation agent noted:

IA1: “*Our clear strategy was to remain open to dialogue, repeated consultation, and ongoing collaboration.*”

Another barrier in this phase was miscommunication within and between organisations, especially when implementation agents were not involved. This fuelled misinformation and resistance among potential users. To counteract this, implementation agents routinely re-engaged stakeholders with accurate information and refocused communication on the intervention’s core goals. Misinterpretation of legal information was particularly common, which led to the introduction of monthly consulting hours with the BIB-Pro Data Protection Officer (DPO).

IA3: “*We have now also organised DPO meetings as it was observed that a lot of incorrect and negative communication was circulating among the DPOs [of potential partner-organisations].*”

Strong government leadership was also essential. NIHDI’s mandate provided credibility and long-term perspectives for organisations, a key asset in Belgium’s fragmented health system, where regional variations often delay implementation. As one agent noted:

IA1: “*We have a complex country with intricate structures, where multiple levels of government must reach agreements—and each parliament must give its approval. All of that takes time, and so people wait [before acting] until they have certainty.*”

Beyond the outer context and bridging factors, inner context constructs also emerged prominently. Consistent with the EPIS constructs ‘Leadership’ (Inner Context) and ‘Individual Characteristics’, implementation agents noticed that organisational decision-makers and department heads acted as key leaders influencing adoption. Their engagement determined how the platform was prioritised within organisations and whether resources were allocated to support its use. Decision-makers weigh the perceived benefits against the potential drawbacks, often advised by DPO’s, when deciding whether to adopt the intervention. These decisions may be based on objective considerations, but also on subjective judgment.

Over time, two adoption pathways emerged. Initially, some organisations adopted the platform based on its preventive mission, a decision driven more by values. Later, the introduction of a financial incentive appealed to more practical and objective reasons, leading to faster adoption.

IA1: “*We can also feel that this financial incentive is becoming a real facilitator—even in organisations that were previously more hesitant.*”

At the operational level, department heads and early adopters acted as influential peers whose support could motivate others or persuade more sceptical colleagues. In this sense, recognition of the intervention’s added value acts as a facilitator.

IA2: “*If you don’t see the added value, or if you don’t understand why it’s so important… then, of course, you definitely won’t do it.*”

Conversely, resistance to change, digital unfamiliarity, workload concerns, discomfort discussing psychosocial issues, or worries about data privacy acted as barriers in this phase.

IA4: “*And yes, the resistance to change is enormous. So, in general, that’s what you’re up against: something new. Something unfamiliar. Something digital.*”

To overcome these challenges, implementation agents provided targeted support, such as training on psychosocial screening and digital use, clear information about data handling, and support in developing internal agreements on platform use to ensure workload feasibility.

**Figure 7:**
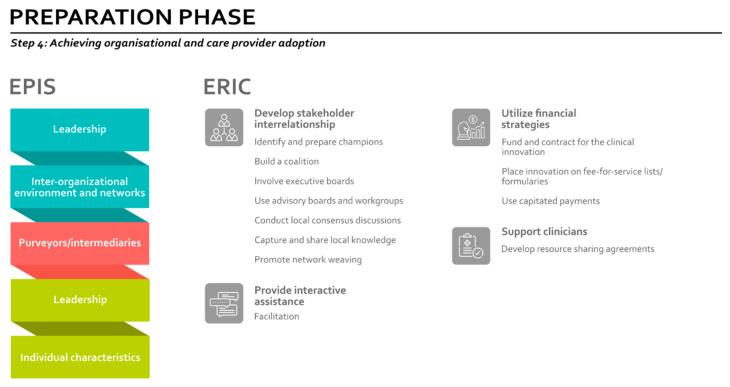
EPIS constructs and ERIC strategies applied in step four of the Preparation phase.

In summary, besides the BIB-Pro team acting as facilitators through interactive assistance and developing shared agreements to support care providers, other strategies employed at this stage focused on developing stakeholder interrelationships and making use of financial strategies (Figure 7).

#### 3.2.5. Step 5: Preparing the Organisation to Use the Intervention

At this stage, the focus shifted to the Inner context once decision-makers signed the necessary formal agreements. Consistent with the EPIS constructs ‘Leadership’ and ‘Individual Characteristics’, successful preparation within (health) care organisations depended strongly on involved executives (e.g., CEO, DPO, IT manager) who endorsed the initiative, and end users (e.g., midwives, gynaecologists, social services, psychologists) who would integrate the platform into daily practice.

Implementation agents, acting as Purveyors/Intermediaries, therefore aimed to ensure that all stakeholders were informed and engaged. They supported organisations’ internal infrastructural and procedural changes needed to integrate the platform into daily workflows. They fostered a supportive implementation culture through training, briefings, and close follow-up.

IA4: “*That’s already a conclusion I can draw, proximity is crucial—or even necessary—to properly follow up and to roll it out in a high-quality way.*”

Implementation agents reported that having a designated internal coordinator(s) who ensures consistent communication, is responsible for coordinating the deployment, ensures follow-up and serves as the central point of contact, is a key implementation strategy for facilitating uptake within the organisation and aligns with the EPIS construct “Quality and Fidelity Monitoring/Support”. As one agent stressed:

IA5: “*We need to have an internal leader, because without one, we can’t break through the walls.*” She gave a hospital as an example: “*Three lead midwives act as intermediaries. In fact, we’ve established a dedicated forum—a meeting, you might say—where frontline staff are consulted. These aren’t top-down decisions that simply have to be implemented.*”

Consistent with the EPIS constructs ‘Organisational Characteristics’ and ‘Staffing Processes’, successful deployment also required adequate infrastructure and resources—such as appropriate workspaces, hardware, interoperable IT systems, and sufficient staffing—and alignment with existing workflows. Lack of clear referral pathways, for example, acted as a barrier:

IA3: “*If a hospital offers midwife consultations but patients aren’t yet systematically or adequately referred to those consultations, you’re stuck—because that’s their internal flow, not something that’s necessarily linked to [the Born in Belgium Pro platform].*”

**Figure 8:**
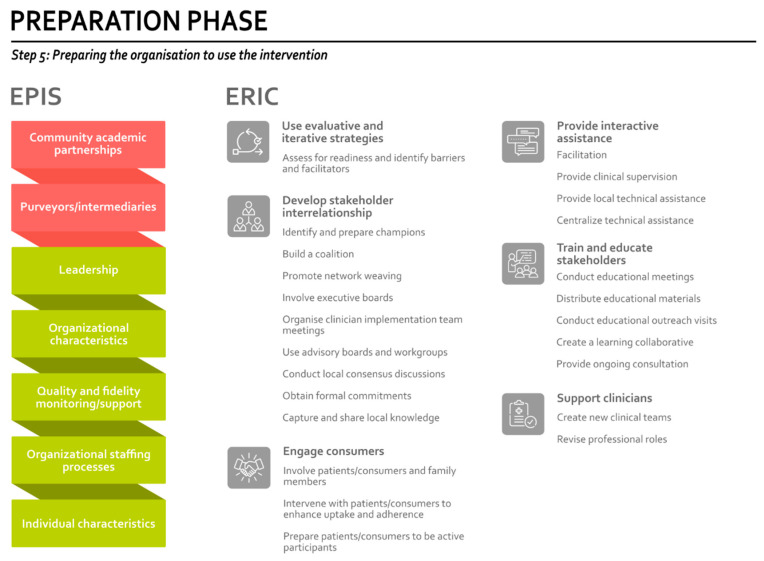
EPIS constructs and ERIC strategies applied in step five of the Preparation phase.

In this final preparation step (Figure 8), the team applied a combination of ERIC strategies covering six strategies-classification. They, for example, offered interactive assistance through facilitation and local technical support. To strengthen engagement, they fostered stakeholder relationships by preparing internal champions (or coordinators), involving executive leadership, holding consensus discussions, and securing formal commitments. Finally, they invested in stakeholder training through educational meetings.

### 3.3. Implementation

Implementation reflected the active use of the BIB-Pro platform in practice and unfolded along two interconnected tracks: (1) internal integration within organisations (inner context), and (2) broader adoption across community care networks (outer context).

The first implementation track focused on organisational adoption. This stage was shaped by the Preparation phase. Once the platform was in use by care providers, implementation agents (Purveyors/Intermediaries) offered ongoing support through continued training and meetings to address questions, troubleshoot technical and operational issues, and adapt strategies based on field feedback. These continuous feedback loops helped maintain alignment between the platform’s design and real-world use, reinforcing its ‘Innovation/EBP Fit’.

IA6: “*Be available to answer any questions that arise*”

Despite reaching the Implementation phase, a major barrier was the limited number of implementation agents to systematically monitor platform use across all organisations and regions. Organisations with internal coordinators who tracked internal usage, identified local needs, and maintained contact with implementation agents were better able to ensure continuity. When internal coordinators discontinued their role, usage dropped. To support continuous monitoring, the agents developed a dashboard that provided both organisations and agents with insight into platform activity, usage levels, and referral behaviour. This evaluative tool strengthened ‘Quality and Fidelity Monitoring/Support’.

Consistent with the EPIS construct ‘Inter-organisational Environment and Networks’, the second implementation track focused on network-level adoption, extending the platform’s implementation to the organisations’ surrounding networks (i.e., community care networks, including health or social care providers and organisations). It became particularly relevant due to barriers such as weak or absent networks, limited network mapping, and low awareness of local services. Moreover, no or partial uptake within a network undermined collaboration and demotivated independent care providers, who often saw little return on their investment of time and effort.

IA1: “*Hospitals rarely work transmurally or maintain care alliances, and they often lack knowledge of regional service offerings.*” She also noted that: “*A strong network is a facilitator [for implementation]; a weak one is a barrier.*”

Especially since one of the platform’s functionalities relies on accurate and up-to-date local network databases, incomplete or missing regional databases in one region made it difficult for providers to use the platform’s referral system. This was described by one of the implementation agents as a care provider’s barrier regarding the use of the platform:

IA5: “*They began using the platform without proper [databases for referral for the] follow-up, and the same issues kept recurring. As a result, they lost motivation to continue [using the platform]*”. Implementation agents provided a solution to that: “*We [implementation against] created our own network regional database centred on [Hospital X], which is now integrated into the platform.*”

To strengthen inter-organisational collaboration and ensure formal commitment, agents facilitated local interdisciplinary working groups that shared ownership of the implementation process, aiming to develop a common care pathway and a framework for collaboration. Approximately five to ten working groups were held, led by local healthcare stakeholders (Leadership) and supported by implementation agents. This approach fostered engagement, promoted shared ownership, and resulted in regional cooperation and referral databases, thereby supporting broader adoption and integrated use of the platform within networks.

IA1: “*We revised our strategy to emphasise regional reach: before rolling out within a single hospital, we ensured that all organisations in the surrounding area were aware of the project, identified active local partners, and fostered connections across the care continuum.*”

**Figure 9:**
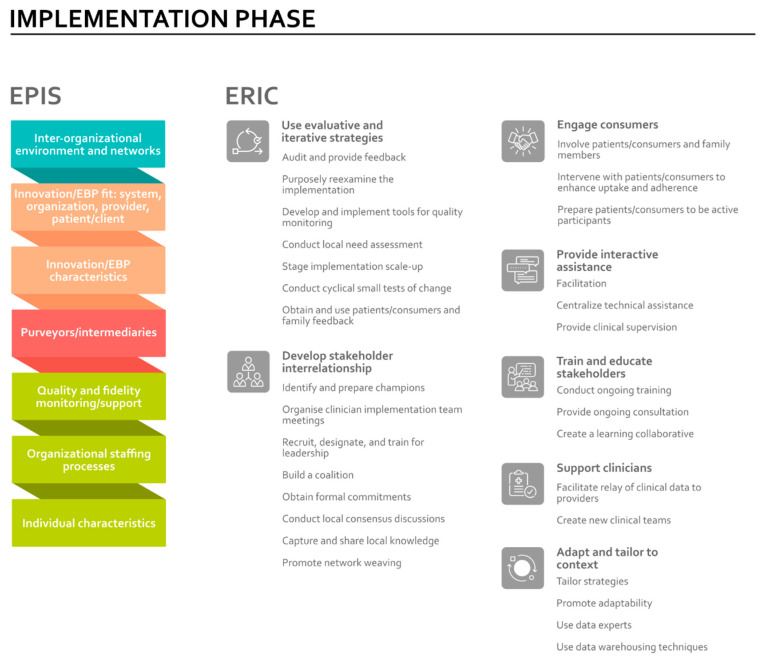
EPIS constructs and ERIC strategies applied during the Implementation phase.

To summarise, the main strategies used during the Implementation phase were mainly focused on building strong stakeholder interrelationships, providing interactive assistance and using evaluative and iterative strategies. The latter included auditing platform usage, providing feedback from and to users, periodically re-examining implementation efforts, developing tools for monitoring (such as a built-in dashboard), organising quality monitoring systems, conducting regional needs assessments, and engaging in small tests of change to refine the process over time. Figure 9 provides an overview of all implementation strategies applied.

### 3.4. Sustainment

The Sustainment phase focused on the continued use and structural embedding of the BIB-Pro platform within organisations and the healthcare system. Like implementation, it required ongoing follow-up, stakeholder meetings, and monitoring to ensure integration into daily practice.

Several enablers supported the long-term integration of the platform. In the Outer Context, the constructs ‘Funding/Contracting’ and ‘Service Environment/Policies’ were key. Continuous funding across all phases was essential to sustain the technical platform development and provide incentives for adoption among users. In parallel, sustained institutional and policy support was needed to align the platform with evolving national priorities and data regulations.

Within this policy context, some organisations raised concerns about the institutional legitimacy of data controllership for a large-scale, government-supported health data platform. Although the non-profit organisation responsible for the platform fully complied with GDPR and international data security standards, these concerns did not relate to deficiencies in data protection measures—such as privacy policies, data-sharing agreements, security protocols, or data protection impact assessments—but rather to the absence of an explicit legislative framework. According to these organisations, data controllership for initiatives of this public scale should be formally assigned to a governmental authority through legislation, rather than delegated to a non-profit entity. One implementation agent explained this as follows:

IA1: “*So at the moment, the legal entity underpinning the platform is a non-profit organisation. And they believe that it is not appropriate for a non-profit to be the entity responsible for data processing. For a project like this, they feel it should be regulated by the government. Within a legislative framework, the government should be the data controller.*”

The governmental authorities are in the process of addressing this issue through legislative adjustments, but this is a lengthy and time-consuming process. According to the agents, legislative frameworks lagging behind digital innovation risk stakeholders’ disengagement if regulation does not evolve in parallel with practical implementation. One agent emphasised the importance of policy and governmental support when wanting to upscale implementation at the national level:

IA1: “*I think there is a difference between successes in implementation [at a smaller level] and successes in national implementation. Success in implementation means collaboration and an electronic, IT-based system that can communicate, that follows the right standards and respects all [European data] regulations—that is the basis of a good implementation. But if you also want to implement nationally, you need that extra layer to be able to demonstrate full government support and the existence of legislation. […] At the same time, it is of course possible to implement without legislation, but then it will remain at a smaller scale.*”

At the Innovation level, the constructs ‘Innovation/EBP Fit’ and ‘Innovation Characteristics’ were central. Important was the commitment to further co-create intervention development (Innovation Factor), which ensured that the platform remains adaptable to the evolving needs of care providers. Implementation agents (Purveyors/Intermediaries) introduced a yearly user survey to gather feedback on needs for improvement, which informed a development roadmap created with the DPO and technical partner. Planned improvements were presented at the annual user meeting to foster transparency and engagement. Furthermore, to maintain scientific validity and practical relevance, a five-year roadmap was established to update psychosocial vulnerability indicators and related care pathways through expert panels—strengthening ‘Quality and Fidelity Monitoring/Support’.

**Figure 10:**
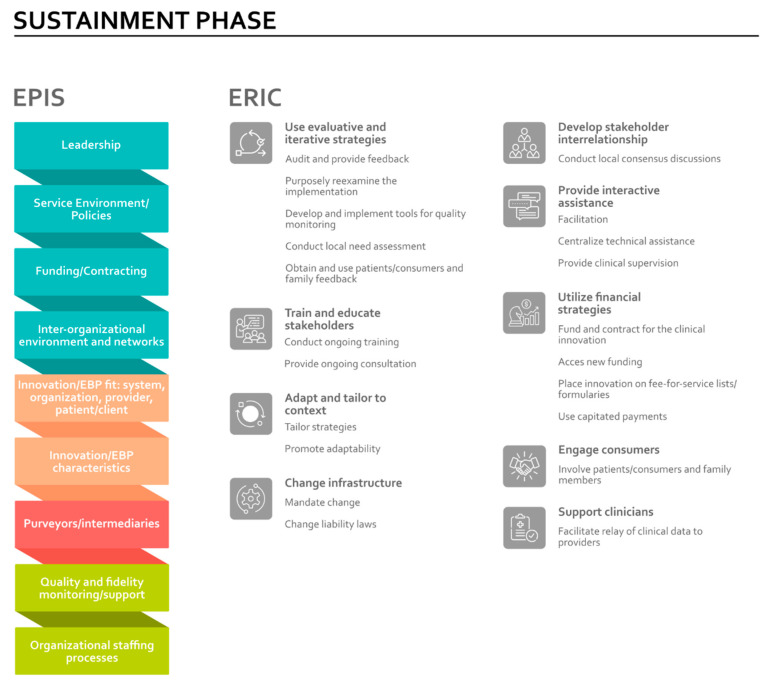
EPIS constructs and ERIC strategies applied during the Sustainment phase.

During the Sustainment phase, strategies (Figure 10) applied included: building stakeholder relationships, providing interactive assistance, and iterative evaluation, while also requiring infrastructure and financial strategies, including mandated change, liability adjustments, or new reimbursement models.

### 3.5. Key Attributes Implementation Agents

During the interviews, it became clear that implementation agents hold some essential attributes. Agents should be accessible, collaborative, motivators, listen actively to stakeholder needs, have patience, be resilient, have skills in addressing resistance and fears, and additionally, be flexible in communication (email, phone, in-person, online). Their role as a bridge between practice and policy is vital, while recognising that certain organisational or governmental constraints are fixed and beyond their influence. Co-creation and collaboration remained central, not only with care providers and organisations but also within the BIB-Pro team itself. This was reinforced by regular internal process evaluations.

IA7: “*We need to convey in our attitude that we are always present, and if there’s even the slightest question, we are there.*”

IA1: “*We followed the philosophy of a government, not that of a business. In a company, you focus on your own growth and advancement, whereas as a public institution, you must ensure that you serve everyone.*”

IA3: “*In recent years, we’ve conducted extensive process evaluations [….] we continually assess whether we’re still operating correctly ourselves, which isn’t always easy.*”

## 4. Discussion

This study provided insight into how a shared digital innovation was implemented in a real-world, cross-sectoral perinatal care setting. Using the EPIS framework and ERIC taxonomy, this study examined the implementation process, contextual determinants, and implementation strategies of the BIB-Pro CDSS in perinatal care.

### 4.1. Implementation Process of a Shared Platform

The BIB-Pro implementation process progressed through all four EPIS phases. The Exploration phase identified the appropriate intervention, the Preparation phase set up and planned the rollout, the Implementation phase launched the platform and ensured continued use, and the Sustainment phase concentrated on monitoring and adjusting at the Innovation, Inner, and Outer context levels. Covering all phases was key to the success of the BIB-Pro implementation. As Moulin et al. emphasise in their systematic review, focusing solely on the Implementation phase is insufficient, as thorough planning during the Exploration and Preparation stages is crucial for later success [22]. Similarly, as Gu. al. stress in their systematic review of CDSS in mental health helpline services, neglecting one or more phases of the system development cycle (i.e., analysis, design, development, implementation, and evaluation) increases the risk of unsuccessful implementation [24].

Still, going through all EPIS phases does not guarantee success; understanding the contextual factors that influenced the BIB-Pro implementation is therefore also essential.

### 4.2. Main Contextual Determinants

When synthesising the findings, three key determinants of the implementation process were identified: (1) the coordinating role of implementation agents as bridging actors, (2) stakeholder involvement and collaboration, and (3) system-level governance conditions, including leadership, policy, and funding. These findings align with those of Schmitt et al.’s systematic review, which identified leadership engagement, internal implementation leaders, compatibility, available resources, external change agents, champions, relative advantage, and key stakeholders as key determinants influencing implementation processes [5].

#### 4.2.1. Implementation Agents as Bridging Actors

Implementation was not a purely technical rollout but required continuous alignment between policy, organisations, and care providers. This coordination was driven by implementation agents (i.e., the BIB-Pro team), who acted as acting as bridging actors, connecting stakeholders, facilitating communication, and maintaining momentum throughout the process. They applied multiple implementation strategies, including ongoing support and follow-up (e.g., providing interactive assistance and developing stakeholder interrelationships), to facilitate both organisational adoption and broader scale-up. While their role was most prominent during the Preparation phase, it remained important throughout the Implementation and Sustainment phases.

These findings support prior research highlighting the importance of intermediaries in implementation processes [25,26]. According to Lengnick-Hall et al., bridging factors not only connect outer and inner contexts but can extend their influence across multiple phases, potentially spanning the entire implementation process [27]. In this study, the BIB-Pro team’s contribution went beyond supporting implementation: they also acted as intervention developers, directly shaping and adapting the content of the intervention, while simultaneously fulfilling the functions of a bridging actor. By co-developing the intervention with stakeholders, they ensured that innovation characteristics and fit with practice remained central from Preparation through Sustainment. Maintaining this consistent value–innovation fit leads to more successful innovation implementation [22].

Consistent with Santema et al. [9], who studied CDSS implementation in inter-organisational contexts, our findings underscore the importance of an orchestrating actor in coordinating communication, alignment, and technical support across organisations, reflecting the critical role of implementation agents in enabling inter-organisational CDSS implementation.

In addition to their coordinating role, implementation agents’ approach and competencies represent a fundamental part of implementation. We found that regular, tailored follow-up, adjusted to the needs of each organisation, was essential for driving and maintaining change. This has been confirmed by Alagoz et al. (2018)’s systematic review, which states that information sharing or audit and feedback alone, without close follow-up, are not enough [28]. However, overly dependent collaboration—where an organisation that relies heavily on external partners and staff outside their own organisation—can undermine sustainability [29]. Therefore, overall management should remain within organisations, while external partners adopt a supportive role.

Equally important were the agents’ personal competences such as collaboration, openness, resilience, patience, and adaptability. These qualities, especially the collaborative attitude, are important given the frequent use of stakeholder interrelationship strategies seen in the BIB-Pro implementation process. This aligns with Bührmann et al. (2022)’s review [30]. Taken together, these findings highlight that implementation success is not only dependent on the selection of strategies but also on the actors delivering them. We therefore underscore the importance of incorporating the attributes of implementation agents into future implementation frameworks and strategies.

#### 4.2.2. Multi-Level Stakeholders’ Involvement and Collaboration

While Schmitt et al.’s systematic review identified “key stakeholders” as one of the determinants most frequently influencing implementation processes, it remained unclear how these stakeholders were defined and differentiated, as many studies did not specify their roles or levels of involvement, making it difficult to understand their specific contributions to implementation [5]. Our findings provide further insight into this by showing that stakeholder involvement is not only important but also highly differentiated across roles and system levels. They illustrate that stakeholders’ involvement, with actors fulfilling different roles within the implementation process, extended beyond a single organisational setting and across multiple care settings and system levels, reflecting the cross-sectoral and inter-organisational nature of the BIB-Pro platform.

At the macro level, the National Institute for Health and Disability Insurance (NIHDI), as policy actor and funder, was involved from the outset and supported the development and broader uptake of the platform. At the meso level, organisational leadership appeared to shape adoption, with executive managers involved in the initial decision to adopt the platform. The presence of internal coordinators supported its integration into routine practice by guiding organisational adjustments, monitoring use, and facilitating communication between staff and the BIB-Pro team. When such structures were in place, implementation appeared to progress more smoothly into later phases, whereas their absence was associated with challenges in maintaining use. At the micro level, individual characteristics, such as peer influence, affected both adoption and continued use.

Besides the involvement of multiple actors, implementation within a cross-sectoral and inter-organisational care context also appeared to rely on collaboration between them. During the Exploration and Preparation phases, collaboration was essential for building consensus and shaping the platform. In the Implementation phase, it enabled dissemination and rollout, while in the Sustainment phase, it supported ongoing platform improvement and structural changes at both organisational and policy levels. This is in line with Santema et al., a study specifically examining CDSS implementation in an inter-organisational primary care context, which describes collaboration as a continuous, iterative process sustained through shared principles, coordination by an orchestrating actor, and ongoing co-creative experimentation spanning both development and implementation phases.

Taken together, our findings are consistent with previous research indicating that early stakeholder involvement, alignment around a shared vision, and multilevel engagement are important for implementing complex interventions in real-world, multi-organisational healthcare contexts [5,9,29,31,32,33,34].

#### 4.2.3. System-Level Conditions Shaping Implementation

Implementation depended not only on local efforts, but also on alignment with broader system-level conditions (i.e., governance, infrastructures, legislation and policy), which enabled or constrained progress. Policy support and funding were essential to initiate and sustain the implementation, while financial incentives contributed to broader uptake at later stages. In particular, the integration of the BIB-Pro platform into the national Perinatal Program in 2024 [35], allowing health care providers to receive reimbursement for offering care through the platform, further strengthened sustainment and facilitated wider adoption.

In contrast, uncertainty around data controllership within a non-profit framework emerged as a key barrier. This concern was addressed through collaboration between governance actors and implementation agents, using a proactive approach that included implementation strategies such as providing consultative support (e.g., DPO meetings), developing formal implementation blueprints, and advocating for infrastructural changes (i.e., legislative adjustments).

More broadly, structural legislative adjustments remain necessary to facilitate data-sharing in countries governed by multiple regional authorities—a crucial step for continued scale-up and long-term sustainability. A study in Switzerland illustrates this challenge, where nationwide adoption of digital health innovations is complicated due to tiered governance and 26 cantons with different health laws [36]. This demonstrates why policymakers and commissioners must be engaged throughout the entire implementation process. Consistent with this, Derkens et al. (2025)’s systematic review recommends that policymakers establish a strategy for the sustainable integration of digital health solutions into healthcare systems, including clear governance and quality-control mechanisms, clarification of liability and medico-legal rules, guidance on CDSS use, and adequate funding to support development and implementation [37].

### 4.3. Implementation Strategies Used

While mapping the implementation process helped to understand the main contextual determinants that shaped the BIB-Pro implementation process, it is equally important to understand the specific strategies that were applied to move the process forward. These strategies were not applied sequentially but were repeatedly used throughout the implementation process. Using the ERIC taxonomy, we found that nearly all 73 strategies were employed. Five clusters were most prominent across the entire process: developing stakeholder interrelationships, using evaluative and iterative strategies, engaging consumers, training and educating stakeholders, and providing interactive assistance. Other clusters, such as adapting and tailoring to context, changing infrastructure, supporting clinicians, and utilising financial strategies, were less frequent but nonetheless essential.

While these clusters represent the overarching strategies, their relative importance varied across the EPIS phases. In the Exploration phase, evaluative and iterative strategies (e.g., needs assessment) and stakeholder interrelationships (e.g., advisory boards, consensus discussions) ensured local relevance and early buy-in. The Preparation phase consisted of engaging consumers, training and educating stakeholders, and providing interactive assistance, together with iterative planning and relationship-building. During the Implementation phase, clinician support, interactive assistance, stakeholder interrelationships, and continuous monitoring promoted adoption, collaboration, and sustained use. Finally, during the Sustainment phase, ongoing evaluation and stakeholder engagement enabled continuous improvement of both the CDSS and the implementation process.

Overall, we found that the strategies used are quite like other studies [8,28,37,38,39,40]. The BIB-Pro implementation process applied several core strategies that were identified in a systematic review as being successful in at least 75% of cases, including educational activities, site diagnostic activities, facilitation, and quality improvement strategies [8]. Furthermore, given its multi-organisational and multi-level implementation process, BIB-Pro’s scale-up depended on upscaling strategies. In line with Leeman et al. (2022) [38], these included dissemination, stakeholder engagement, training and technical assistance, policy development, resource mobilisation, and continuous monitoring and evaluation. Similarly, USA national-level recommendations on CDS optimisation emphasised strategies such as developing stakeholder interrelationships—for example, engaging federal leadership for CDSS standards development and maturation or developing a multistakeholder CDS learning community to inform usability. Their recommendations also highlighted the use of evaluative and iterative strategies (e.g., disseminating best practices; measuring CDS usage) and changing infrastructure (e.g., promoting financing and measurement to accelerate CDS adoption; and creating a CDS legal framework) [39], all approaches that were likewise applied in the BIB-Pro implementation process.

Evidence from the perinatal care field further supports this perspective. A qualitative study in the perinatal setting using a collaborative care program highlighted the importance of strategies such as building coalitions, identifying and preparing champions, adapting physical structures, revising professional roles and ensuring external funding as key enablers for implementation [40]. These findings, focusing on an implementation process in a single care setting, resonate closely with our results, which further extend insights by reporting more on inter-organisational implementation across different settings.

### 4.4. Limitations and Strength

The key strength of this study includes its comprehensive insight into CDSS implementation processes, contextual determinants, and implementation strategies within a cross-sectoral, inter-organisational care context. By examining implementation across multiple settings and system levels, this study contributes to a limited body of literature that moves beyond single-organisation perspectives. In addition, the combined use of the EPIS framework and ERIC taxonomy, which, to our knowledge, has never been applied in Belgium and only once in perinatal care [40]. Further strengths include BIB-Pro’s multi-setting, multi-level scale-up, which allowed understanding of inter-organisational dynamics and outer-context influences. Another strength lies in the method: deductive coding at the construct level allowed for precision and reproducibility, structuring results across phases for clarity, and triangulating with supplementary data for robustness. Finally, by focusing on implementation agents as bridging actors between inner and outer context, this study provides a comprehensive overview of how implementation processes unfold in real-world settings, integrating insights across system levels and implementation phases.

Some limitations should be acknowledged. First, the deductive nature of the analysis, while ensuring consistency with established frameworks, may have limited the identification of unexpected themes and may have contributed to an overstructured interpretation of the data. Nevertheless, one inductively derived theme did emerge: the identification of key attributes of implementation agents, extending current frameworks. Second, the study relied primarily on self-reported data from implementation agents, which introduces potential biases, including social desirability bias and hindsight bias. Participants may have presented the implementation process more coherently or positively in retrospect than it unfolded in practice. In addition, the prior professional relationship between the researcher and participants may have influenced responses and introduced potential bias in data collection and interpretation. Third, although implementation agents act as a bridging factor between inner and outer context, our findings reflect their perspective and may not capture those of other stakeholders, such as frontline healthcare professionals, policymakers, or end users. The absence of negative-case sampling and the limited inclusion of less engaged or critical stakeholders may have reduced the ability to identify contrasting or disconfirming evidence. Furthermore, triangulation was mainly based on internal sources (interviews and implementation documents), without external validation. Finally, although the settings involved in the implementation were diverse, this still limits the generalisability of the findings.

## 5. Conclusions

This explorative case study examined the implementation of the BIB-Pro CDSS within a real-world, cross-sectoral perinatal care context, providing a structured overview of the implementation process, contextual determinants, and strategies. The implementation process was shaped by bridging functions, multilevel stakeholder involvement, inter-organisational collaboration, and system-level support. Implementation agents appeared to play a central coordinating role by linking policy, organisational contexts, and clinical practice, while supporting alignment and continuity throughout the process. In addition, strategies such as developing stakeholder interrelationships, providing interactive assistance, and applying evaluative and iterative approaches were consistently identified across phases and may have contributed to organisational adoption, continued use, and broader dissemination of the platform. This study provides a first insight into the implementation of a digital innovation within an inter-organisational perinatal care context. Future research should examine how these insights can be adapted to other healthcare settings.

## Data Availability

The data presented in this study are available on request from the corresponding authors. The data are not publicly available due to privacy or ethical restrictions.

## Supplementary Materials

The following supporting information can be downloaded at: https://www.mdpi.com/article/10.3390/healthcare14111508/s1. Supplementary File S1: Semi-structured interview guide; Supplementary File S2: ERIC taxonomy structured form.

## Abbreviations

The following abbreviations are used in this manuscript:

BIB-Pro	Born in Belgium Professionals
CDSS	Clinical Decision Support System
CFIR	Consolidated Framework for Implementation Research
DPO	Data Protection Officer
EHR	Electronic Health Record
EBP	Evidence-Based Practice
EPIS	Exploration, Preparation, Implementation, Sustainment
ERIC	Expert Recommendations for Implementing Change
GDPR	General Data Protection Regulation
IA	Implementation Agent
IT	Information Technology
NIHDI	National Institute for Health and Disability Insurance
