# Supplementary material for: Process and Strategies for Implementing an Antenatal Psychosocial Clinical Decision Support System Within an Inter-Organisational Care Context: The Born in Belgium Professionals Platform
Source: Healthcare (Basel). 2026 May 29;14(11):1508. doi: 10.3390/healthcare14111508 (PMC13257003; doi:10.3390/healthcare14111508)
Supplement: Supplementary file 1 [file healthcare-14-01508-s001.zip › Additional File S1_Interview Guide.pdf]

Supplementary File S1

Interview Questions Based on the EPIS Framework

Thank you for your time. The purpose of this interview is to understand the implementation process of BIB — how your team approached it, your experiences, strategies, and challenges.

Preparation Phase

1. Can you explain the process of how a CDSS platform like Born in Belgium Professionals was implemented across different organisations?
2. What were the main steps in the preparation phase of the platform within the organisations?
3. Which tools (e.g., training materials, manuals) and strategies (e.g., workshops, communication plans) were developed to support the implementation?
4. How did the team prepare organisations and users for the introduction of BIB-Pro?
5. What obstacles did you encounter during the preparation phase, and how were these resolved?
6. What worked well during this phase; what were the key facilitators?

Implementation Phase

7. What were the main steps in the implementation phase of the platform within the organisations?
8. Which implementation strategies were applied to encourage users and organisations to adopt the platform, and how effective were they?
9. What forms of resistance emerged during implementation, and how were they addressed?
10. What were the main facilitators during the implementation phase?
11. How was it ensured that the platform was integrated into existing workflows within the organisations?
12. In your opinion, what would the ideal organizational structure look like to support successful implementation, and which elements are essential?

Sustainment Phase

13. Which strategies and measures were used to ensure that the platform remains useful in the long term?
14. What are the main lessons learned during the implementation process?
15. What are the key lessons learned to ensure that an organisation continues to use the platform over time?
